# 5′ capped and 3′ polyA-tailed sgRNAs enhance the efficiency of CRISPR-Cas9 system

**DOI:** 10.1007/s13238-018-0552-5

**Published:** 2018-06-04

**Authors:** Wei Mu, Yongping Zhang, Xutong Xue, Lei Liu, Xiaofei Wei, Haoyi Wang

**Affiliations:** 1 State Key Laboratory of Stem Cell and Reproductive Biology, Institute of Zoology, Chinese Academy of Sciences, Beijing, 100101 China; 2 University of Chinese Academy of Sciences, Beijing, 100049 China; 3 Department of Hematology, Aerospace Center Hospital, Aerospace Clinical Medical College, Peking University, Beijing, 100049 China; 4 Hebei University, Baoding, 071002 China; 5 Beijing Cord Blood Bank, Beijing, 100044 China

**Dear Editor,**

The clustered regularly interspaced short palindromic repeats (CRISPR)-associated (Cas) system is an adaptive immune system in a variety of bacteria and archaea (Terns and Terns, 2011). The most commonly used *Streptococcus pyogenes* type II CRISPR-Cas9 system consists of Cas9 nuclease and two short RNAs, crRNA and tracrRNA, which can be linked together forming one chimeric single guide RNA (sgRNA) (Jinek et al., 2012). Guided by sgRNA, Cas9-sgRNA complex can generate DNA double strand breaks (DSB) at specific genomic loci (Jinek et al., 2012; Cong et al., 2013; Mali et al., 2013). Cas9 with mutations at the catalytic RuvC and HNH domains loses the endonuclease activity (indicated “dCas9”), while maintains its DNA binding capability (Gilbert et al., 2013). DCas9 fused with transcription effectors, such as VP64, P65-HSF1 and KRAB can be guided to specific promoters by sgRNA, enabling regulation of gene transcription (Gilbert et al., 2013, 2014).

RNA structures and sequences that can be recognized by specific RNA binding proteins were incorporated into sgRNA backbone to enable more efficient and versatile effector recruiting such as synergistic activation mediator (SAM) system (Konermann et al., 2014), Casilio system (Cheng et al., 2016) and CRISPR-Display (Shechner et al., 2015). In addition, chemical modifications such as 2′-O-methyl 3′-phosphorothioate (MS) or 2′-O-methyl 3′ H-thioPACE (MSP), which have been shown to enhance siRNA stability (Deleavey and Damha, 2012; Eckstein, 2014), were applied to sgRNA as well as crRNA and tracrRNA (Hendel et al., 2015; Rahdar et al., 2015), leading to improved gene editing efficiency. Despite all these advances, chemical synthesis of long RNA oligos with these modifications is challenging and expensive. With current length limitation of RNA chemical synthesis technologies, it is difficult to generate sgRNA with extra sequences or structures. Here, we established a modification method to improve the stability of *in vitro* transcribed (IVT) sgRNA and therefore the efficiency of CRISPR-Cas9 system, which can be adapted by any lab with basic molecular biology experience.

We first amended different modifications to the backbone of IVT sgRNA based on previously published literatures describing structures stabilizing RNA in cells (Bergman et al., 2007; Chapman et al., 2014). To mimic the RNA structure stabilized by LSM family proteins (Bergman et al., 2007), polyA tract was added to the 5′ end of sgRNA (indicated as polyA-sgRNA). Inspired by the structure of Dengue virus subgenomic flaviviral RNAs (sfRNAs) (Chapman et al., 2014), stem loop II (SLII), stem loop IV (SLIV) and stem loop at the 3′ terminal (3′SL) of Dengue virus sfRNA were added to the 5′ or both ends of the sgRNA. We also mimicked the mRNA structure by adding 5′ cap and 3′ polyA tail to sgRNA (CTsgRNA). The schematic structures of these differently modified sgRNAs were shown in Figs. 1A and S1A. To test their stability, we delivered equal amounts of *AAVS1* sgRNAs with different structures into K562 cells by electroporation, and analyzed their quantities in the cells by qPCR at different time points (Figs. 1B and S1B). Among all the modified forms, only the CTsgRNA had better performance. The residual quantity of CT modified *AAVS1* sgRNA was 3.1-fold of unmodified sgRNA two hours post electroporation, and twelve hours later the unmodified *AAVS1* sgRNA declined almost to none while the CT modified sgRNA still persisted (Fig. 1B). Other structures did not improve the persistence of sgRNA (Fig. S1B).

**Figure 1:**
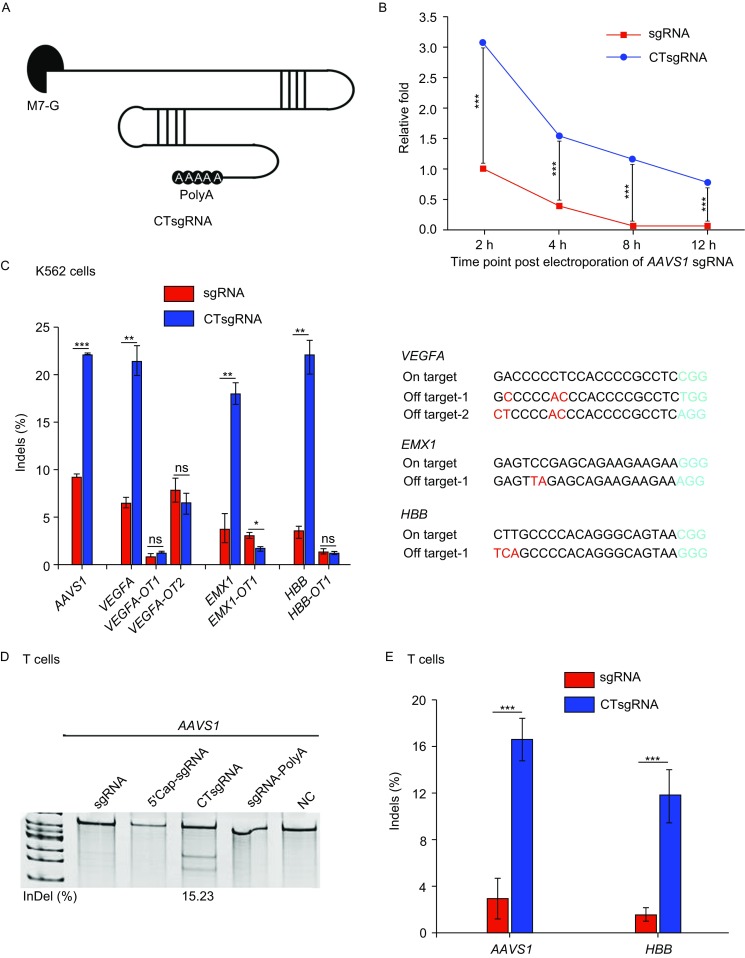
**5′ cap and 3′ polyadenylation modified sgRNA enhanced genome editing efficiency of CRISPR-Cas9 system in human K562 cell line and primary T cells**. (A) Schematic structure of CT modified sgRNA. (B) Stability of unmodified and CT modified *AAVS1* sgRNA in K562 cells. 10 µg unmodified or CT modified *AAVS1* sgRNA were electroporated into two million K562 cells. The quantity of sgRNA was measured by qPCR at different time points, and Ru6B was used as an internal control. Each data point depicts the relative abundance of sgRNA in electroporated cells at each time point (mean ± SD, *n* = 3). (C) Gene editing on-target and off-target efficiency of indicated sgRNA in K562 cells, quantified by TIDE analysis. OT: Off-target. (D and E) Gene editing efficiency in stimulated primary T cells. 10 µg *AAVS1* sgRNA modified with different structures and 10 µg Cas9 mRNA were electroporated into stimulated T cells. Gene editing frequencies were analysed by Surveyor assay (D) and TIDE sequencing (E). Bars represent average indels frequency ± SD, *n *= 3. *P < 0.05, **P < 0.01, ***P < 0.001, *P* values were calculated by employing an unpaired *t*-test comparing with the values from control group

Next, we evaluated whether CT modification on sgRNA led to a higher genome editing efficiency. We delivered each *AAVS1* sgRNA structure together with *in vitro* transcribed Cas9 mRNA into K562 cells by electroporation and analyzed genome disruption frequencies by Surveyor assay (Fig. S2A). Consistent with the improved stability, CTsgRNA led to higher insertion or/and deletion (indel) frequencies (26.90%) compared to control sgRNA (15.29%), while the sgRNAs with other structures had an editing efficiency lower than control (Fig. S2A). Improved gene editing efficiency using CTsgRNAs was also obtained at the *VEGFA*, *EMX1*, *HBB* and *PD1* loci in K562 cells, with indel frequency quantified using tracking of indels by decomposition (TIDE) analysis (Figs. 1C and S3), Surveyor assay (Fig. S2B) and chip-based QuantStudio 3D digital PCR (Fig. S4).

As CT modification on sgRNA led to higher on-target editing efficiency, we evaluated whether it also affected the off-target activity. We tested three sgRNAs with well-defined off-target sites, and found that the CTsgRNAs induced similar level of off-target indel frequencies when compared to the unmodified sgRNA (Fig. 1C). However, we cannot exclude the possibility that CTsgRNA caused higher mutation rates at other off-target sites. When sgRNAs with different structures and Cas9 mRNA were delivered into human primary T cells, genome editing induced by unmodified *AAVS1* sgRNA was almost undetectable, while CTsgRNAs resulted in editing frequencies of 15.23% (Fig. 1D). Neither 5′ cap nor the 3′ polyA tail modification alone was able to induce indels more efficiently than unmodified sgRNAs in primary T cells (Fig. 1D). Similar results were obtained at the *HBB* loci (Fig. 1E). Taken together, these results showed that the CT modification enhanced sgRNA intracellular stability, improved genome editing efficiency in K562 and human primary T cells.

We further applied CTsgRNA to transcription regulation (Fig. 2A), using *OCT4*, *NANOG* and *KLF4* as target genes. For each gene, four sgRNAs targeting the promoter region (200 bp upstream of transcription start site) were used as a pool. We co-delivered dCas9-P65HSF1 expressing plasmid and *OCT4*sgRNA pool with or without CT modification into K562 cells. The CT modified sgRNA pool was able to activate endogenous *OCT4* for 62.2-fold, while the unmodified pool only 2.3-fold. When dCas9-P65HSF1 mRNA was used instead of plasmid, CT modified sgRNA pool was able to increase endogenous *OCT4* expression 138-fold, while the unmodified sgRNA pool only 11-fold (Fig. 2B). Similarly, CT modified sgRNA pool led to significant gene activation at both *KLF4* and *NANOG* loci, while unmodified sgRNA pool had minor or no effect (Fig. 2C). When the sgRNA pools activating three genes were applied simultaneously, we observed all three genes were significantly activated only in samples treated with CTsgRNAs (Fig. S5A). These results demonstrated that the CT modified sgRNA enhanced CRISPR-dCas9 mediated activation of endogenous genes in K562 cells.

**Figure 2:**
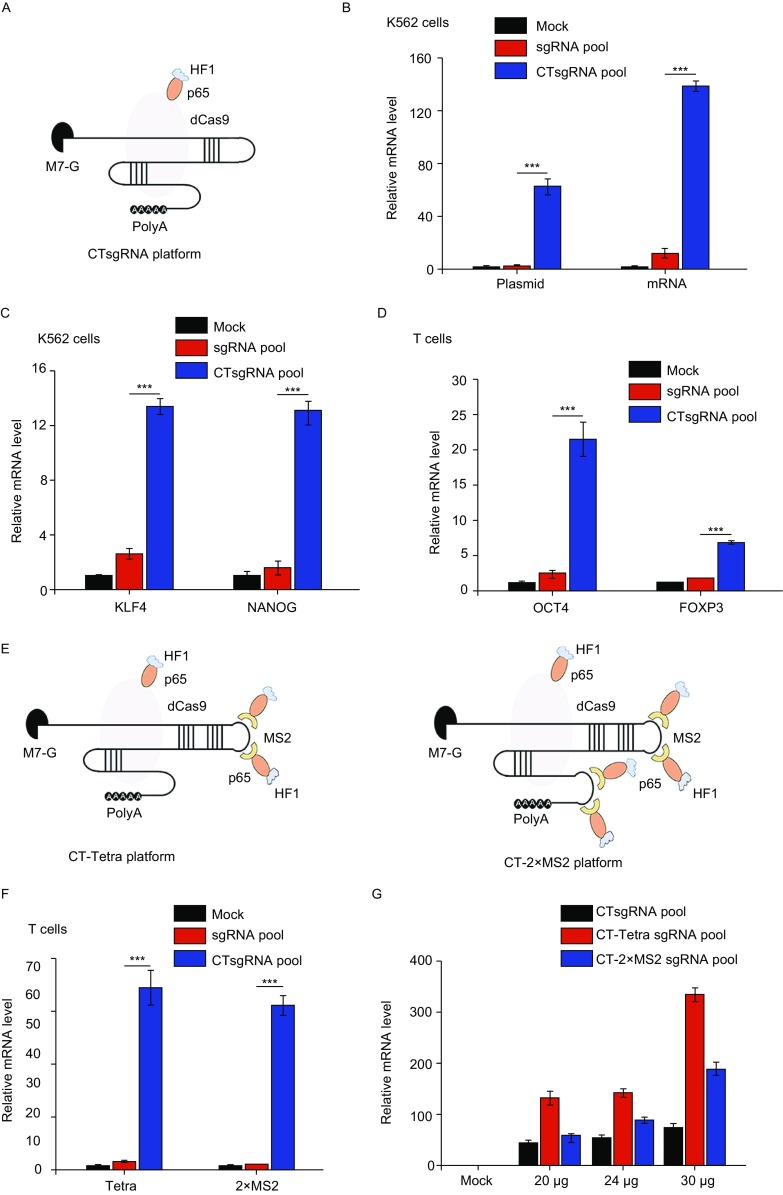
**CT modified sgRNAs enhanced endogenous gene activation in both K562 cells and primary T cells**. (A) Schematic structure of CTsgRNA gene activation platform. (B) The activation of *OCT4* in K562 cells, using dCas9-P65HSF1 expressing plasmid or mRNA. (C) The activation of *KLF4* and *NANOG* in K562 cells. (D) The activation of *OCT4* and *FOXP3* in stimulated primary T cells. (E) Schematic structures of CT-Tetra and CT-2×MS2 gene activation platforms. (F) CT modification on sgRNA containing MS2 binding sites improved the activation of *OCT4*. 10 µg dCas9-p65HSF1 mRNA, 5 µg MS2-P65HSF1 mRNA and 5 µg indicated sgRNA pool were electroporated into 3 million stimulated T cell. (G) Comparison of three gene activation platforms using different quantity of reagents. For CTsgRNA platform the ratio of dCas9-p65HSF1 mRNA and CTsgRNA pool is 1:1. For CT-Tetra or CT-2×MS2 platform the ratio of dCas9-p65HSF1 mRNA, MS2-P65HSF1 mRNA and CT modified sgRNA pool is 1:1:2. For all the gene activation experiments, gene expression was quantified by qPCR using *GAPDH* as control. Bars represent average expression level of each gene in three replicates ± SD. *P < 0.05, **P < 0.01, ***P < 0.001, *P* values were calculated by employing an unpaired *t*-test comparing with the values from control group

We next tested whether CTsgRNA can be applied to efficiently activate endogenous genes in human primary T cells. We chose *OCT4* and *FOXP3* as target genes. Delivery of dCas9-P65HSF1 mRNA with the unmodified sgRNA pool barely activated target genes, while CT modified sgRNA pool induced 22-fold and 7-fold improvement at *OCT4* and *FOXP3* mRNA level, respectively (Fig. 2D). Based on flow cytometry analysis using FOXP3 antibody, the mean fluorescence intensity increased more than 2-fold (Fig. S5B). Since the gene activation system was expressed transiently, the *FOXP3* positive cells decreased with time (Fig. S5C).

To further improve the performance of CT modified sgRNA and establish an efficient endogenous gene activation platform in primary T cells, we applied the CT modification to two sgRNA structures (sgRNA1.1 and sgRNA2.0) descried previously (Konermann et al., 2014), (1) sgRNA1.1: one copy of MS2 hairpin aptamer was incorporated into the tetraloop of the sgRNA backbone, indicated as Tetra sgRNA; (2) sgRNA2.0: two copies of MS2 hairpin aptamers were incorporated into the tetraloop and stem loop 2 of sgRNA backbone, indicated as 2×MS2 sgRNA (Fig. 2D). We delivered dCas9-P65HSF1 mRNA, MS2-P65HSF1 mRNA and unmodified or CT modified sgRNAs with different structures: *OCT4* Tetra or 2×MS2 sgRNA pool simultaneously into primary CD3^+^ T cells. Schematic structures of these two complexes were shown in Fig. 2E. Unmodified *OCT4* Tetra and 2×MS2 sgRNA pools did not activate endogenous *OCT4* efficiently. Remarkably, both activated *OCT4* expression up to 60-fold after CT modification (Fig. 2F). To further improve the endogenous gene activation in human primary T cells, we optimized the proportion of different components. DCas9-P65HSF1 mRNA, MS2-P65 mRNA and CT modified Tetra sgRNA pool at the ratio of 1:1:2 led to the best activation efficiency in CD3^+^ T cells (Fig. S6A). We further optimized the total amount of reagents used, and achieved more than 300 fold activation of *OCT4* gene in primary T cells using CT modified Tetra sgRNA pool (Fig. 2G), with reasonable cell viability (Fig. S6B). Thus, the CT modified Tetra sgRNA system consisted of three components further improved the endogenous gene activation in primary T cells.

In this study, we demonstrated that: (I) sgRNA stability was enhanced by adding 5′ cap and 3′ polyA tail; (II) CT-modified sgRNA mediated higher level of genome disruption than unmodified sgRNA both in human cell line and primary T cells; (III) CT modified sgRNA with MS2 binding sites mediated efficient activation of endogenous gene expression.

By combining CT modification with sgRNA containing MS2 binding sites, we achieved efficient activation of endogenous genes in human primary T cells. The capability of controlling the expression of endogenous genes will help us better study the functions of different genes and the transcription networks in various primary cells. We have shown that genes important to T cell function, such as *FOXP3*, can be efficiently activated using our method, providing exciting possibilities of changing cell fate by regulating gene expression. Efficient activation of endogenous gene in primary cells holds great potential for both basic research and therapeutic applications.

## Footnotes

We would like to thank Junning Wei and Yi Yang (Beijing Cord Blood Bank) for their help in preparing the cord blood samples. This work was supported by the National Natural Science Foundation of China (Grant No. 31471215), Strategic Priority Research Program of the Chinese Academy of Sciences (No. XDA16010205), National Key Research and Development Program of China (No. 2016YFA0101402), and National High-tech R&D Program (863 Program) (No. 2015AA020307). H. Wang is supported by the “Young Thousand Talents Plan”.

W. Mu concept and design, collection and assembly of data, date analysis and interpretation, manuscript writing; Flow cytometry were conducted by Y. Zhang. X. Xue: collection of data; Templates for *in vitro* transcription was made by L. Liu. X. Wei prepared and provided essential reagents to the experiments. H. Wang: concept and design, manuscript writing, and final approval of the manuscript.

Wei Mu, Yongping Zhang, XuTong Xue, Lei Liu, Xiaofei Wei and Haoyi Wang declare that they have no conflict of interest. All procedures followed were in accordance with the ethical standards of the responsible committee on human experimentation (institutional and national) and with the Helsinki Declaration of 1975, as revised in 2000 (5). All donors of umbilical cord blood (UCB) units have provided informed consent.

## Electronic supplementary material

Below is the link to the electronic supplementary material.

Supplementary material 1 (PDF 943 kb)
