# Supplementary material for: 5′ capped and 3′ polyA-tailed sgRNAs enhance the efficiency of CRISPR-Cas9 system
Source: Protein Cell. 2018 Jun 4;10(3):223–8. doi: 10.1007/s13238-018-0552-5 (PMC6338620; doi:10.1007/s13238-018-0552-5)
Supplement: Supplementary file 1 — Supplementary material 1 (PDF 943 kb) [file 13238_2018_552_MOESM1_ESM.pdf]

# SUPPLEMENTARY DATA

## Methods

### Human T cell isolation and culture

Fresh umbilical cord blood (UCB) units obtained from healthy volunteer donors who had provided informed consent from the Beijing Cord Blood Bank (Beijing, China). Peripheral blood mononuclear cells (PBMCs) were separated by density gradient centrifugation with human mononuclear cells separation medium 1.077 (DongFangHuaHui, 25710). $CD3^+$ T cells were isolated using the EasySep human T cell enrichment kit (Stemcell Technologies, 19051), activated and expanded with CD3/CD28 Dynabeads (Gibco, 11163D) at a bead to T cell ratio of 1:1 according to the manufacturer's instructions. UCB-derived $CD3^+$ T cells were cultured in X-vivo15 medium (Lonza, 04-418Q) supplemented with 5% (v/v) heat-inactivated fetal bovine serum (Gibco, 10091-148) in the presence of 100 IU/mL recombinant human IL-2. All cells were cultured at 37 °C in 5 % $CO_2$ atmosphere.

### Cell culture and electroporation

K562 cells were maintained in RPMI 1640 (Gibco, C11875500BT) supplemented with 10 % FBS, 100 mg/ml streptomycin and 100 units/ml penicillin (Gibco, 15140-122). K562 cells were electroporated with the 4D-Nucleofector X and core Unit (Lonza) and SF cell line 4D-Nucleofector Kit (Lonza, V4XC-1024). For gene editing experiments in K562 cells: 10 μg unmodified or differently modified sgRNA and 10 μg Cas9 mRNA

were electroporated into 1 million cells using program FF120. For gene activation experiments in K562 cells: 10 μg unmodified or CT modified sgRNA and 10 μg dCas9-P65 mRNA or 1 μg dCas9-P65 expressing plasmid were electroporated into 1 million cells using program FF120.

T cells were activated for three days with CD3/CD28 Dynabeads before electroporation. T cells were electroporated using the Lonza Nucleofector 4D (Lonza) and P3 Primary Cell 4D-Nucleofector Kit (Lonza, V4XP-3024). For gene editing experiments in T cells: 10 μg unmodified or modified sgRNA and 10 μg Cas9 mRNA were electroporated into 1 million cells using program EO115. For gene activation experiments in T cells: 10 μg unmodified or modified sgRNA and 10 μg dCas9-P65 mRNA or as the dose in figure legend were electroporated into 3 million cells using program EO115.

**_In vitro_ transcription**

Guide sequences of sgRNAs and oligo primers used in this study were listed in Table S1. The sequence of DNA templates for T7-sgRNA PCR were listed in Table S2. The T7-sgRNA PCR product was recovered and used as template for *in vitro* using MEGA shortscript T7 kit (Ambion, AM1354) and mMESSAGE mMACHINE® T7 Ultra Kit for CT modification (Ambion, AM1345). RNAs were purified with MEGAclear columns (Ambion, AM1908) and eluted with RNANase-free water.

**Surveyor assay and Tracking of Indels by Decomposition (TIDE) sequencing**

The levels of genomic disruption of *AAVS1*, *VEGFA*, *EMX1*, and *HBB* in K562 cell

line or primary T cells was determined by Surveyor assay using Surveyor mutation detection kit (Integrated DNA Technologies, Inc). The percentage of target disruption was quantified by densitometry and calculated as described (Guschin et al., 2010). The PCR products are also sequenced for TIDE analysis using specially designed software that provided as a simple web tool (available at http://tide.nki.nl). The PCR primers used for the amplification of target locus and sequencing are listed in Table S1.

**QuantStudio® 3D Digital PCR**

Digital PCR is a method of quantitative analysis of sample nucleic acids based on single-molecule template PCR amplification without the use of standard curves. Primers and probes were designed as previously described (Mock et al., 2016). Genomic DNA was extracted by using EasyPure Genomic DNA Kit (Transgene, EE101). Mixture consisted of 900nM PD1-dPCR-F, 900nM PD1-dPCR-R， 2x QuantStudio® 3D digital PCR master mix (Thermo, A26358), 200nM VIC-Insensitive probe-MGB, 200nM FAM-sensitive probe-MGB and sample gDNA was loaded onto chips (Thermo, A26316 ) using the QuantStudio® 3D Digital PCR Chip Loader. The chips were sealed and loaded onto ProFlex™ 2x Flat PCR System (Applied Biosystem) and cycled as following parameters: 96℃ for 10 min, followed by 39 cycles of 52℃ for 2 min and 98℃ for 30 sec and then extension at 60℃ for 2 min. After cycling, the chips were measured by QuantStudio™ 3D Digital PCR Instrument. The data was analyzed by AnalysisSuite™ at https://china.apps.thermofisher.com/quantstudio3d/.

**Quantitative PCR**

Total RNA was extracted with the Trizol reagent (Life Technologies, 15596-026 ). 1 μg RNA of each sample was used for reverse transcription with TransScript-Uni One-Step gDNA Removal and cDNA Synthesis Supermix Kit (TransGen Biotech, AU311). QPCR reactions were performed using qPCR Master Mix (Takara, QPK-212) in CFX96 real-time detection system (Bio-Rad). Housekeeping gene *GAPDH* was used as internal control. The qPCR primers are listed in Table S1.

**Flow cytometry**

CytoFLEX (Beckman Coulter Inc) was used to perform fluorescent expression analysis. Cells were harvested 48 hours, 72 hours and 96 hours post electroporation and stained with mouse anti-human FOXP3 antibody (eBioscience, 11-4776-42) for 1 hour in the dark refrigerator.

**Statistics**

Statistical significance was analyzed by unpaired *t*-tests using GraphPad Prism 5 with *P*-value considered as ***$P < 0.001$; **$P < 0.01$; *$P < 0.1$.

**REFERENCE**

Guschin, D.Y., Waite, A.J., Katibah, G.E., Miller, J.C., Holmes, M.C., and Rebar, E.J (2010) A Rapid and General Assay for Monitoring Endogenous Gene Modification. In Engineered Zinc Finger Proteins: Methods and Protocols, J.P. Mackay, and D.J. Segal, eds. (Totowa, NJ: Humana Press), pp. 247-256.

Mock, U., Hauber, I. and Fehse, B. (2016) Digital PCR to assess gene-editing frequencies (GEF-dPCR) mediated by designer nucleases. *Nat. Protoc*., 11, 598

**Supplementary Figures**

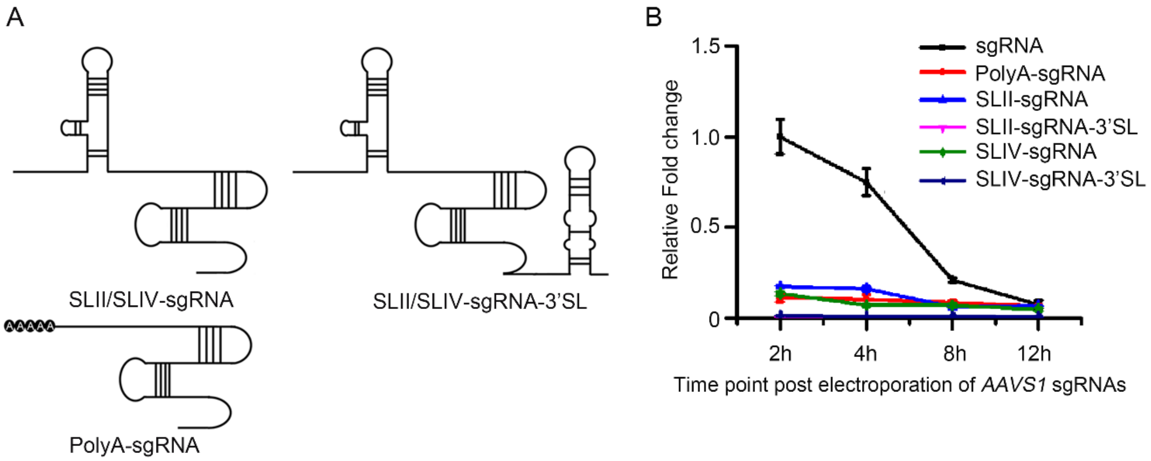


**Figure S1** The stability of differently modified sgRNA structures in K562 cell line.

(A) Schematic structures of differently modified sgRNAs. SLII: stem loop II of Dengue virus sfRNA, SLIV: stem loop IV of Dengue virus sfRNA, 3’SL: stem loop at 3’end of Dengue virus sfRNA. Poly A: 55 consecutive A bases. (B) Stability of modified *AAVS1* sgRNAs in K562 cells. 2 million K562 cells were electroporated with 10 µg IVT *AAVS1* sgRNA with indicated structures. The quantity of sgRNAs were measured by qPCR at different time points, and *Ru6B* was used as an internal control. Each data point depicts the relative abundance of sgRNA in electroporated cells at each time point (Mean ± SD, n = 3).

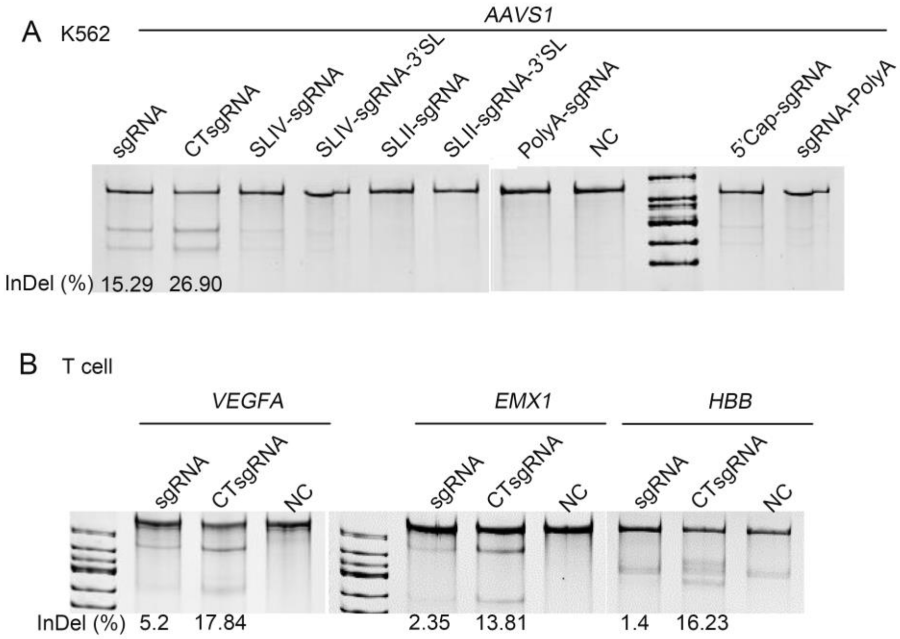


**Figure S2** Indel frequencies of sgRNA with different modifications in K562 and primary CD3$^+$ T cells as measured by Surveyor assay. (A) Gene editing efficiency at *AAVS1* site mediate by different sgRNA structures in K562 cells. (B) CT modification enhanced gene disruption at *VEGFA*, *EMX1* and *HBB* sites in K562 cells. Percentage of gene disruption is indicated under each panel.

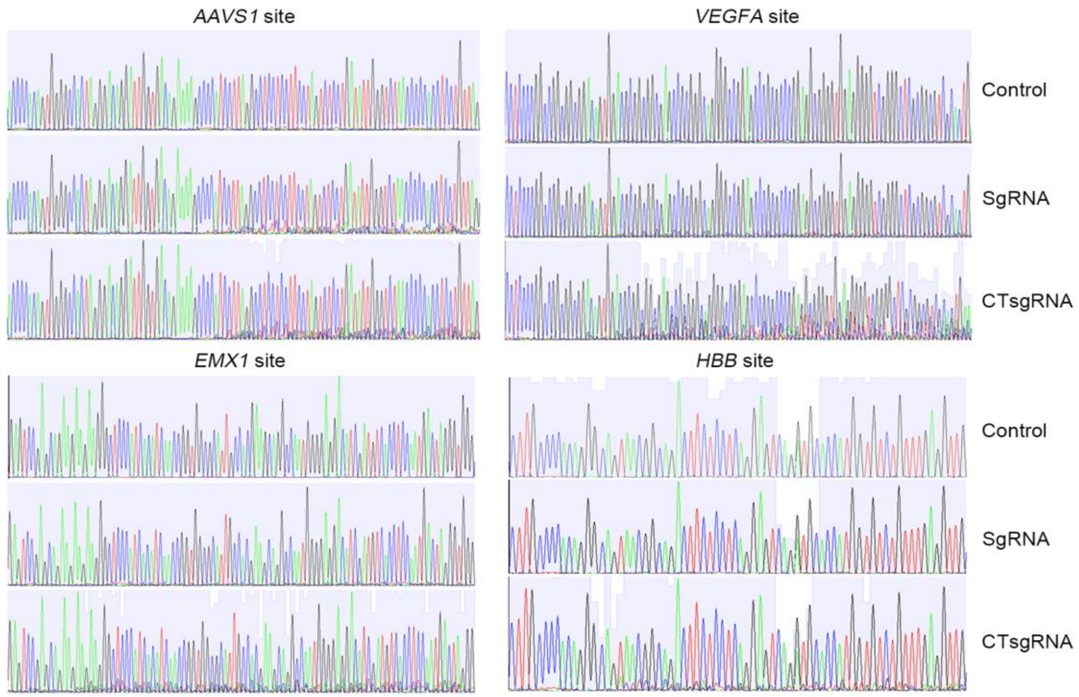


**Figure S3** Sequencing profile of TIDE related to **Figure 1C.**

| Target | Sample | Copies/μl | CI Copies/μl | Precision |
|---|---|---|---|---|
| FAM | K562 -1 | 1541.2 | 1511.6 -- 1571.5 | 1.96% |
| VIC | K562 -1 | 1540.7 | 1511.1 -- 1571 | 1.96% |
| FAM | K562 -2 | 865.46 | 846.49 -- 884.86 | 2.24% |
| VIC | K562 -2 | 873.47 | 854.37 -- 892.99 | 2.23% |
| FAM | PD1 sgRNA-1 | 1370.2 | 1344.2 -- 1396.7 | 1.93% |
| VIC | PD1 sgRNA-1 | 1382.2 | 1356 -- 1408.8 | 1.93% |
| FAM | PD1 sgRNA-2 | 1398.8 | 1372.3 -- 1425.9 | 1.94% |
| VIC | PD1 sgRNA-2 | 1404.6 | 1378 -- 1431.8 | 1.93% |
| FAM | PD1 CTsgRNA-1 | 1461 | 1432.9 -- 1489.7 | 1.96% |
| VIC | PD1 CTsgRNA-1 | 1521.3 | 1492.2 -- 1551 | 1.95% |
| FAM | PD1 CTsgRNA-2 | 1467.1 | 1439.4 -- 1495.3 | 1.92% |
| VIC | PD1 CTsgRNA-2 | 1530.3 | 1501.6 -- 1559.5 | 1.91% |

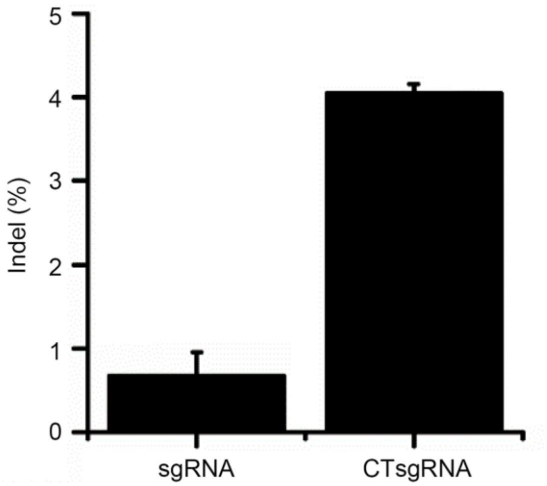


**Figure S4.** CT modification enhanced gene editing efficiency at *PD1* loci in K562 cells measured by QuantStudio® 3D Digital PCR. Left: copy numbers of FAM or VIC positive signal in the gDNA of control and gene-edited K562 cells. Right: Gene editing efficiency based on copy number analysis. Gene editing efficiency = (1-FAM/VIC)%. Bars represent average editing efficiency in two replicates ± SD

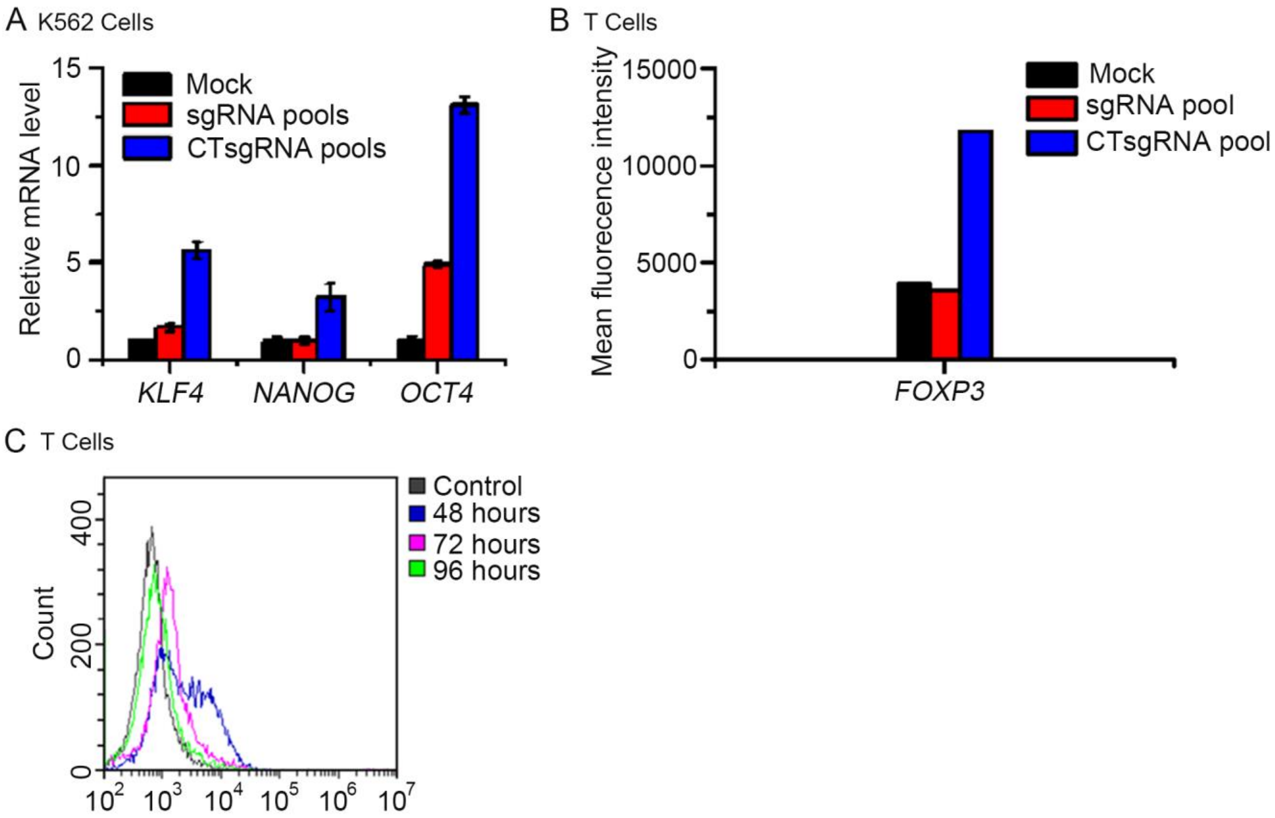


**Figure S5** CT modification improved gene activation in K562 and primary T cells. (A) CTsgRNA platform enabled simultaneous activation of three genes in K562 cells. The expression of each gene was measured by qPCR with *GAPDH* as an internal control. Bars represent average mRNA level of each gene in three replicates ± SD. (B) The protein level of endogenous *FOXP3* was represented by mean fluorescence intensity, measured 48 hours post electroporation by flow cytometry. (C) Expression of *FOXP3* protein in T cells at different time points post electroporation, analyzed by flow cytometry.

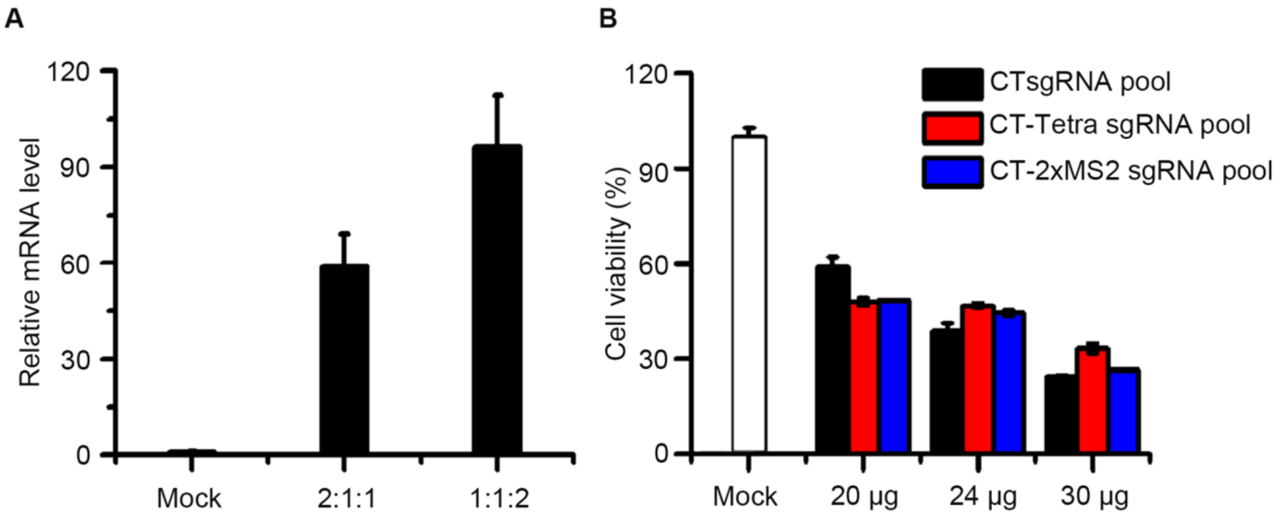


**Figure S6** Optimization of gene activation method in T cells.

(A) The optimal ratio of CT-Tetra platform components in activating endogenous *OCT4* gene. 3 million stimulated human primary $CD3^+$ T cells were electroporated with 20 μg total amount of RNA. The ratio of dCas9-p65HSF1 mRNA, MS2-P65HSF1 mRNA and CT modified tetra OCT4 sgRNA pool is 2:1:1 or 1:1:2. The mRNA level of endogenous *OCT4* was quantified by qPCR with *GAPDH* as an internal control. Bars represent average mRNA level of *OCT4* in three replicates ± SD. (B) Cell viability of three gene activation platforms. Different quantity of RNAs of each gene activation platform were delivered into 3 million stimulated human primary $CD3^+$ T cells and cell viability was determined by cell counting 2 days after electroporation. The ratio of dCas9-p65HSF1 mRNA, MS2-P65HSF1 mRNA and CT modified tetra OCT4 sgRNA pool is 1:1:2. Bars represent average cell viability of electroporated cells in three replicates ± SD.

## Supplemental Tables

**Table S1. Sequences of sgRNA guide and DNA oligos used in this study**

| | |
|---|---|
| **Guide sgRNA sequence** | |
| *AAVS1* sgRNA | ACCCCACAGTGGGGCCACTA |
| *VEGFA* sgRNA | GACCCCCTCCACCCCGCCTC |
| *EMX1* sgRNA | GAGTCCGAGCAGAAGAAGAA |
| *HBB* sgRNA | CTTGCCCCACAGGGCAGTAA |
| *OCT4* sgRNA1 | GGCCCCGCCCCCTGGATGGG |
| *OCT4* sgRNA2 | GGGGGGAGAAACTGAGGCGA |
| *OCT4* sgRNA3 | GGTGGTGGCAATGGTGTCTG |
| *OCT4* sgRNA4 | GACACAACTGGCGCCCCTCC |
| *KLF4* sgRNA1 | GCGCGCTCCACACAACTCAC |
| *KLF4* sgRNA2 | AAGGAACGCGCGCCGGCGGC |
| *KLF4* sgRNA3 | ATGGGAGAAGGCGGAGGAAA |
| *KLF4* sgRNA4 | GCAACGATGGAAGGGAGCCT |
| *NANOG* sgRNA1 | GATTAACTGAGAATTCACAA |
| *NANOG* sgRNA2 | TCTAGTTCCCCACCTAGTCT |
| *NANOG* sgRNA3 | TGTCTTCAGGTTCTGTTGCT |
| *NANOG* sgRNA4 | TGATTTAAAAGTTGGAAACG |
| ***In vitro* transcription primers** | |

| | |
|---|---|
| SgRNA Forward primers | TAATACGACTCACTATAGNNNNNNNNNNNNNNNNNNN(20bp target sequence)GTTTAAGAGCTATGCTGGAAAC |
| SgRNA Reverse primers | AAAAGCACCGACTCGGTGCC |
| SL2S-sgRNA Forward primers | TAATACGACTCACTATAGGGCTAAAAGTCAGGTCGGATC |
| SL2S-sgRNA Reverse primers | AAAAGCACCGACTCGGTGCC |
| SL2S-sgRNA-3’SL Forward primers | TAATACGACTCACTATAGGGCTAAAAGTCAGGTCGGATC |
| SL2S-sgRNA-3’SL Reverse primers | GCAGAATTGGCGCACGCGCTA |
| SL4L-sgRNA Forward primers | TAATACGACTCACTATAGTAAAAGAAGTCAGGCCATCAC |
| SL4L-sgRNA Reverse primers | AAAAGCACCGACTCGGTGCC |
| SL4L-sgRNA-3’SL Forward primers | TAATACGACTCACTATAGTAAAAGAAGTCAGGCCATCAC |
| SL4L-sgRNA-3’SL Reverse primers | GCAGAATTGGCGCACGCGCTA |

| | |
|---|---|
| PolyA-sgRNA Forward primer | TAATACGACTCACTATAGGCAATTGGGCCGGCCAAA |
| PolyA-sgRNA Reverse primer | AAAAGCACCGACTCGGTGCC |
| sgRNA-polyA Forward primer | TAATACGACTCACTATAGNNNNNNNNNNNNNNNNNNNGTTTAAGAGCTATGCTGGAAAC |
| SgRNA-polyA Reverse primer | GCAGAATTGGCGCACGCGCTA |
| Tetra sgRNA Forward primer | TAATACGACTCACTATAGNNNNNNNNNNNNNNNNNNNN GTTTAAGAGCTATGCTGG |
| Tetra sgRNA Reverse primer | AAAAGCACCGACTCGGTGCC |
| 2xMBS sgRNA Forward primer | TAATACGACTCACTATAGNNNNNNNNNNNNNNNNNNNN GTTTAAGAGCTATGCTGG |
| 2xMBS sgRNA Reverse primer | AAAAGCACCGACTCGGTGCC |
| DCas9-P65HSF1 Forward primer | TAATACGACTCACTATAGGGAGACCACCATGTACCCATACGATGTTCCAG |
| DCas9-P65HSF1 Reverse primer | TCAATCGATGGAGACAGTGGGGTC |

| | |
|---|---|
| MS2-P65HSF1 Forward primer | TAATACGACTCACTATAGGGAGACCACCATGGCTTCAAACTTTACTCAG |
| MS2-P65HSF1 Reverse primer | TCAGGAGACAGTGGGGTCCTTG |
| SpCas9 Forward primer | TAATACGACTCACTATAGGGAGACCACCATGGACTATAAGGACCACGAC |
| SpCas9 Reverse primer | GCGAGCTCTAGGAATTCTTAC |
| **Genotyping primers for surveyor assays** | |
| *AAVS1*-Forward primer | CTTACCTCTCTAGTCTGTGCTAGC |
| *AAVS1*-Reverse primers | GGATCCTCTCTGGCTCCATCG |
| *VEGFA*-Forward primer | AGAGAAGTCGAGGAAGAGAGAG |
| *VEGFA*-Reverse primer | CAGCAGAAAGTTCATGGTTTCG |
| *EMX1*-Forward primer | GGAGCAGCTGGTCAGAGGGG |
| *EMX1*-Reverse primer | CAGGGCAGAGCCATCTATT |
| *HBB*-Forward primer | TCTGTCTCCACATGCCCAGT |
| *HBB*-Reverse primer | CAGGGCAGAGCCATCTATT |
| *VEGFA*-OT1-Forward primer | CCAGGTGGTGTCAGCGGAGG |
| *VEGFA*-OT1-Reverse primer | TGCCTGGCCCTCTCTGAGTCT |

| | |
|---|---|
| *VEGFA*-OT2-Forward primer | CCAGGTGGTGTCAGCGGAGG |
| *VEGFA*-OT2-Reverse primer | TGCCTGGCCCTCTCTGAGTCT |
| *EMX1*-OT1- Forward primer | CTCTCCTTCAACTCATGACCAGC |
| *EMX1*-OT1- Reverse primer | CTGCACATGTATGTACAGGAGTC |
| *HBB*-OT1- Forward primer | TCCCGTTCTCCACCCAATAG |
| *HBB*-OT1- Reverse primer | GATTTCCAGGCTATGCTTCCA |
| **Primers for TIDE sequence** | |
| *AAVS1* Forward primer | GAGAGCTCAGCTAGTCTTCT |
| *AAVS1* Reverse primer | TTAGAGGTTCTGGCAAGGAG |
| *VEGFA* Forward primer | GTGCGAGCAGCGAAAGCGAC |
| *VEGFA* Reverse primer | CGCCTCGGCGAGCTACTCTT |
| *EMX1* Forward primer | GGAGCAGCTGGTCAGAGGGG |
| *EMX1* Reverse primer | GGGAAGGGGGACACTGGGGA |
| *HBB* Forward primer | TCTGTCTCCACATGCCCAGT |
| *HBB* Reverse primer | CAGGGCAGAGCCATCTATT |
| **Primers for qPCR** | |
| *GAPDH*-Forward primer | ATGACATCAAGAAGGTGGTG |
| *GAPDH*-Reverse primer | CATACCAGGAAATGAGCTTG |

| | |
|---|---|
| *OCT4*-Forward primer | GCTCGAGAAGGATGTGGTCC |
| *OCT4*-Reverse primer | CGTTGTGCATAGTCGCTGCT |
| *KLF4*-Forward primer | CAGTGCCAAAAATGCGACCGAGC |
| *KLF4*-Reverse primer | GACCATGATTGTAGTGCTTTCTGGC |
| *NANOG*-Forward primer | GCAGAAGGCCTCAGCACCTA |
| *NANOG*-Reverse primer | AGGTTCCCAGTCGGGTTCA |
| *SgRNA*- Forward primer | GCTGGAAACAGCATAGCAAG |
| *SgRNA*-Reverse primer | GCAGGGTCCGAGGTATTC |
| *RNU6B*- Forward primer | CTCGCTTCGGCAGCACA |
| *RNU6B* -Reverse primer | AACGCTTCACGAATTTGCGT |
| sgRNA Reverse transcription primer | GTCGTATCCAGTGCAGGGTCCGAGGTATTCGCACTGGATACGACAAAAAAGCACCG |
| Probes and primers for Quantstudio 3D digital PCR | |
| PD1-dPCR-F | ACCTGACCTGGGACAGTTTCC |
| PD1-dPCR-R | GCCAGCCCAGTTGTAGCA |
| FAM-Sensitive probe | AGATCCCACAGGCGCCCTGGC |
| VIC-Insensitive probe | CCGCTCACCTCCGCCTGAGC |

**Table S2. Sequences of the DNA templates for different sgRNA expression cassettes.**

| Name and Description | DNA Sequence |
|---|---|

| | |
|---|---|
| U6 promoter-sgRNA | GGCGCGCCGAGGGCCTATTTCCCATGATTCCTTCATATTTGCATATACGATACAAGGCTGTTAGAGAGATAATTGGAATTAATTTGACTGTAAACACAAAGATATTAGTACAAAATACGTGACGTAGAAAGTAATAATTTCTTGGGTAGTTTGCAGTTTTAAAATTATGTTTTAAAATGGACTATCATATGCTTACCGTAACTTGAAAGTATTTCGATTTCTTGGCTTTATATATCTTGTGGAAAGGACGAAACACCGCAATTGGGCCGGCCCCCCGTCTCGAAAGATCTAGATCTCACCNNNNNNNNNNNNNNNNNNNNGTTTAAGAGCTATGCTGGAAACAGCATAGCAAGTTTAAATAAGGCTAGTCCGTTATCAACTTGAAAAAGTGGCACCGAGTCGGTGCTTTTTTGTTTTAGAGCTAGAAATAGCAAGTTAAAATAAGGCTAGTCCGTAGCGCGTGCGCCAATTCTGCAGACAAATGGC |
| U6 promoter-PolyA-sgRNA | GGCGCGCCGAGGGCCTATTTCCCATGATTCCTTCATATTTGCATATACGATACAAGGCTGTTAGAGAGATAATTGGAATTAATTTGACTGTAAACACAAAGATATTAGTACAAAATACGTGACGTAGAAAGTAATAATTTCTTGGGTAGTTTGCAGTTTTAAAATTATGTTTTAAAATGGACTATCATATGCTTACCGTAACTTGAAAGTATTTCGATTTCTTGGCTTTATATATCTTGTGGAAAGGACGAAACACCGCAATTGGGCCGGCCAAAAAAAAAAAAAAAAAAAAAAAAAAAAAAAAAAAAAAAAAAAAAAAAAAAAAAAACCCCGTCTCGAAAGATCTAGATCTCACCNNNNNNNNNNNNNNNNNNNNGTTTAAGAGCTATGCTGGAAACAGCATAGCAAGTTTAAATAAGGCTAGTCCGTTATCAACTTGAAAAAGTGGCACCGAGTCGGTGCTTTTTTGTTTTAGAGCTAGAAATAGCAAGTTAAAATAAGGCTAGTCCGTAGCGCGTGCGCCAATTCTGCAGACAAATGGC |
| U6 promoter-SLII-sgRNA | GGCGCGCCGAGGGCCTATTTCCCATGATTCCTTCATATTTGCATATACGATACAAGGCTGTTAGAGAGATAATTGGAATTAATTTGACTGTAAACACAAAGATATTAGTACAAAATACGTGACGTAGAAAGTAATAATTTCTTGGGTAGTTTGCAGTTTTAAAATTATGTTTTAAAATGGACTATCATATGCTTACCGTAACTTGAAAGTATTTCGATTTCTTGGCTTTATATATCTTGTGGAAAGGACGAAACACCGCAATTGGGCTAAAAGTCAGGTCGGATCAAGCCATAGTACGGAAAAAACTATGCTACCTGTGAGCCCCGTCCAAGGACGTTAAAAAGATCTCACCNNNNNNNNNNNNNNNNNNNNGTTTAAGAGCTATGCTGGAAACAGCATAGCAAGTTTAAATAAGGCTAGTCCGTTATCAACTTGAA |

| | |
|---|---|
| | AAAGTGGCACCGAGTCGGTGCTTTTTTGTTTTAGAG<br>CTAGAAATAGCAAGTTAAAATAAGGCTAGTCCGTAG<br>CGCGTGCGCCAATTCTGCAGACAAATGGC |
| U6 promoter -SLII-sgRNA-3'SL | GGCGCGCCGAGGGCCTATTTCCCATGATTCCTTCATA<br>TTTGCATATACGATACAAGGCTGTTAGAGAGATAATT<br>GGAATTAATTTGACTGTAAACACAAAGATATTAGTAC<br>AAAATACGTGACGTAGAAAGTAATAATTTCTTGGGT<br>AGTTTGCAGTTTTAAAATTATGTTTTAAAATGGACTA<br>TCATATGCTTACCGTAACTTGAAAGTATTTCGATTTCT<br>TGGCTTTATATATCTTGTGGAAAGGACGAAACACCG<br>CAATTGGGCTAAAAGTCAGGTCGGATCAAGCCATAG<br>TACGGAAAAAACTATGCTACCTGTGAGCCCCGTCCA<br>AGGACGTTAAAAAGATCTCACCNNNNNNNNNNNNN<br>NNNNNNNGTTTAAGAGCTATGCTGGAAACAGCATAG<br>CAAGTTTAAATAAGGCTAGTCCGTTATCAACTTGAA<br>AAAGTGGCACCGAGTCGGTGCCAATTGCATATTGAC<br>GCTGGGAAAGACCAGAGATCCTGCTGTCTCCTCAGC<br>ATCATTCCAGGCACAGAACGCCAGAAAATGGAATGG<br>TGCTGTTGAATCAACAGGTTCTAGATCTTTTTTTGTT<br>TTAGAGCTAGAAATAGCAAGTTAAAATAAGGCTAGT<br>CCGTAGCGCGTGCGCCAATTCTGCAGACAAATGGC |

| | |
|---|---|
| U6 promoter -SLIV-sgRNA | GGCGCGCCGAGGGCCTATTTCCCATGATTCCTTCATATTTGCATATACGATACAAGGCTGTTAGAGAGATAATTGGAATTAATTTGACTGTAAACACAAAGATATTAGTACAAAATACGTGACGTAGAAAGTAATAATTTCTTGGGTAGTTTGCAGTTTTAAAATTATGTTTTAAAATGGACTATCATATGCTTACCGTAACTTGAAAGTATTTCGATTTCTTGGCTTTATATATCTTGTGGAAAGGACGAAACACCGCAATTGTAAAAGAAGTCAGGCCATCACAAATGCCACAGCTTGAGTAAACTGTGCAGCCTGTAGCTCCACCTGAGAAGGTGTAAAAAAGATCCCACCNNNNNNNNNNNNNNNNNNNNGTTTAAGAGCTATGCTGGAAACAGCATAGCAAGTTTAAATAAGGCTAGTCCGTTATCAACTTGAAAAAGTGGCACCGAGTCGGTGCCAATTGAGATCTTTTTTTGTTTTAGAGCTAGAAATAGCAAGTTAAAATAAGGCTAGTCCGTAGCGCGTGCGCCAATTCTGCAGACAAATGGC |
| U6 promoter -SLIV-sgRNA-3’SL | GGCGCGCCGAGGGCCTATTTCCCATGATTCCTTCATATTTGCATATACGATACAAGGCTGTTAGAGAGATAATTGGAATTAATTTGACTGTAAACACAAAGATATTAGTACAAAATACGTGACGTAGAAAGTAATAATTTCTTGGGT |

| | |
|---|---|
| | AGTTTGCAGTTTTAAAATTATGTTTTAAAATGGACTA<br>TCATATGCTTACCGTAACTTGAAAGTATTTCGATTTCT<br>TGGCTTTATATATCTTGTGGAAAGGACGAAACACCG<br>CAATTGTAAAAGAAGTCAGGCCATCACAAATGCCAC<br>AGCTTGAGTAAACTGTGCAGCCTGTAGCTCCACCTG<br>AGAAGGTGTAAAAAAGATCCCACCNNNNNNNNNNN<br>NNNNNNNNNGTTTAAGAGCTATGCTGGAAACAGCAT<br>AGCAAGTTTAAATAAGGCTAGTCCGTTATCAACTTG<br>AAAAAGTGGCACCGAGTCGGTGCCAATTGCATATTG<br>ACGCTGGGAAAGACCAGAGATCCTGCTGTCTCCTCA<br>GCATCATTCCAGGCACAGAACGCCAGAAAATGGAAT<br>GGTGCTGTTGAATCAACAGGTTCTAGATCTTTTTTTG<br>TTTTAGAGCTAGAAATAGCAAGTTAAAATAAGGCTA<br>GTCCGTAGCGCGTGCGCCAATTCTGCAGACAAATGG<br>C |
